# Close relationships with caregivers as protective factor for the mental health and functioning of war-affected Congolese youth

**DOI:** 10.1186/s13031-024-00624-2

**Published:** 2024-10-22

**Authors:** Florian Scharpf, Roos Haer, Tobias Hecker

**Affiliations:** 1 Department of Psychology, Bielefeld University, P. O. Box 100131, 33501 Bielefeld, Germany; 2 Institute for Interdisciplinary Research On Conflict and Violence, Bielefeld University, Bielefeld, Germany; 3 Institute of Political Science, Leiden University, Leiden, Netherlands

**Keywords:** War, Armed conflict, Trauma, Mental health, Youth, Caregivers, Relationship quality, Protective factor

## Abstract

**Background:**

Supportive social connections are a crucial determinant of the mental health and adjustment of youth in conflict-torn regions. Conflict-affected youth face particular risks to their well-being due to high levels of trauma exposure and perpetration of violent acts as members of armed groups and post-conflict discrimination. However, little is known about the possible protective role of close relationships with caregivers in the aftermath of trauma. This study examined whether a higher perceived quality of relationships with caregivers would attenuate the associations between exposure to traumatic experiences and four indicators of adjustment (posttraumatic stress symptoms [PTSS], emotional problems, behavioural problems, criminal behaviour) in a sample of 268 war-affected youth (61.2% male, Mage = 16.31 years) living in Bukavu, Democratic Republic of Congo. More than half of the present sample (56.7%) were former members of armed groups.

**Methods:**

Data were collected using quantitative structured interviews and analyzed through regression models using the PROCESS macro.

**Results:**

Higher cumulative trauma exposure was significantly related to higher levels of PTSS and emotional problems, while more frequent perpetration of war-related violence was significantly related to higher levels of PTSS, behavioural problems, and criminal behavior. The perceived quality of relationships with caregivers significantly moderated the associations between youth’s cumulative trauma exposure and all four outcomes. At higher perceived quality of relationships with caregivers, the associations between trauma exposure and emotional problems, behavioural problems, and criminal behaviour were no longer significant and the association with PTSS was significantly weakened. Higher perceived quality of relationships with caregivers was also directly significantly related to lower levels of mental health problems and criminal behaviour.

**Conclusions:**

The findings suggest that interventions that focus on strengthening relationships with caregivers are crucial for supporting the mental health and functioning of youth who experienced and perpetrated war-related violence.

## Background

In 2022, an estimated number of more than one in every six children worldwide lived in areas affected by armed conflict [47]. Armed conflicts entail numerous immediate and indirect consequences on children and youth’s health, well-being and development, such as death and injury, psychological trauma, displacement, and disruption of families, communities, and civic systems, [27]. Government and non-state actors involved in armed conflicts have increasingly recruited children as child soldiers and exploited them for various roles, perpetrating grave violations of children’s rights. According to UNICEF [58], more than 105,000 children have been recruited as child soldiers between 2005 and 2022. It is likely that the actual number of cases is much higher, however, with approximately 337 million children in 39 conflicts worldwide at risk of recruitment as child soldiers in 2020 [41].

Studies with youth from various conflict-affected settings, including Sierra Leone [5, 7], the Democratic Republic of Congo (DRC; [22, 26]), Uganda [15, 31, 44], Iraq [30], Sri Lanka [28] and Nepal [33, 34], have documented the nature and psychological impact of the experiences of former child soldiers. A systematic review of studies published through 2012 [4] noted very high, albeit varying, rates of posttraumatic stress disorder (PTSD; up to 99%) and internalizing problems such as depression (up to 88%) and anxiety (up to 60%) among former child soldiers. Studies including comparison groups consistently found significantly higher rates of trauma-related disorders among former child soldiers compared to those that were not involved with armed groups [15, 30, 34].

The increased vulnerability of developing mental health problems is due to unique risk factors former child soldiers face during and after their times with armed groups [4]. Cumulative exposure to war-related trauma has been identified as a major risk factor for the mental health of both former child soldiers [4] and conflict-affected youth not involved with armed groups [48]. Findings from conflict-affected samples further suggest that individuals’ repeated exposure to violence is related to a higher risk of engaging in aggressive and violent behaviours themselves [20, 24, 29]. Accounts of former child soldiers indicate that, in addition to experiencing and witnessing horrendous acts of violence, many were also forced to commit such acts themselves, often against members of their own families and communities [31, 46]. Studies observed independent associations between the number of perpetrated acts of war-related violence and higher levels of trauma-related mental health problems [8, 15] as well as aggression [2, 3, 13, 22]. Thus, war-affected youth, particularly those who experienced and perpetrated violence, may be at an increased risk of engaging in criminal behaviours with negative short- and long-term consequences for their functioning.

For war-affected youth, the amount and quality of ties with their immediate social environment, e.g., family, friends, and community members, are crucial determinants of their health and functioning [6, 33]. However, their traumatic experiences, actual or assumed involvement in the perpetration of violence, trauma-related symptomatology, and proneness to aggressive and violent behaviour render youth vulnerable to experiencing discrimination and stigmatization by kin and community members. This discrimination and stigmatization can, in turn, exacerbate mental health problems and maladjustment [12, 49]. For instance, longitudinal research with former child soldiers in Sierra Leone found that perceived discrimination and stigma by family and community in the post-conflict phase were associated with increased levels of depression, anxiety, and post-traumatic stress symptoms (PTSS) over time [2, 3, 5, 7]. Perceived discrimination by family and community also mediated associations between perpetration of violence and increased hostility and between being raped and stronger depressive symptoms [2, 3].

From a socio-ecological perspective, relationships with caregivers represent proximal interactive processes that can mitigate the impact of adversity stemming from more distal levels, e.g., the community and society, on children’s development [57]. The term “caregivers” can refer to any adults who ensure the well-being of a child, including biological parents and relatives, but also unrelated members of the community. Importantly, caregivers continue to be key figures for individuals’ well-being in adolescence and adulthood [54, 60]. Higher quality relationships with caregivers may contribute to a youth’s resilience in multiple ways, such as by fostering a sense of safety and belonging through secure attachments, by supporting their ability to regulate emotions and manage stress, and by providing material and instrumental support [9, 52]. Having close relationships with caregivers can be a crucial source of resilience for youth who experienced war-related atrocities, especially for youth involved with armed groups who were deprived of their caregivers’ support in a crucial stage of their development. Accordingly, perceived acceptance and support by family and community members after returning from armed groups were shown to predict improved mental health and well-being of former child soldiers in Sierra Leone [2, 3, 5, 7].

Several studies conducted in non-conflict settings found that higher levels of perceived quality of relationships with caregivers buffered associations between youth’s exposure to community stressors, including violence and negative peer influences, and their mental health problems [19, 42] as well as own engagement in violent behaviours [18, 36]. To date, however, few studies directly examined whether the quality of relationships with caregivers moderates the impact of trauma exposure on the mental health of conflict-affected youth. Limitations of available studies include lack of consideration of the youth’s own exposure to violence [32], examining only PTSS as an outcome [53], relying on parents’ reports of youth’s mental health problems [51] or focusing on youth who had moved to a politically stable high-income setting [56]. Notably, none of these studies considered engagement in criminal behaviours as an indicator of adjustment and no study included former child soldiers, who are at increased risk of psychopathology and adjustment problems due to perpetration of war-related violence. Increased trauma-related psychopathology and psychosocial dysfunctioning, rejection by one`s social environment, and engagement in criminal behaviours can increase individuals’ risk of getting involved with armed groups (again), thereby perpetuating a cycle of violence [35, 49]. Therefore, a better understanding of the role of caregivers in shaping the adjustment of war-affected youth, particularly those with a history of perpetrating violence, could be crucial to support the re-integration of these youth and contribute to peacebuilding efforts in conflict-ridden regions.

The present study aimed to examine whether the perceived quality of the relationships with caregivers moderated associations between cumulative exposure to war-related traumatic experiences and four indicators of adjustment (PTSS, emotional problems, behavioural problems, criminal behaviour) in a sample of conflict-affected youth in Eastern DRC, including former child soldiers. The violent conflict in DRC, which has been ongoing since 1996, has involved clashes between the Congolese army, supported by a peacekeeping mission of the United Nations (MONUSCO), and over 100 different armed rebel groups. Since its beginning, the conflict has claimed approximately six million victims and displaced about seven million people within the country [25]. Violent attacks and human rights violations against civilians, particularly children, have been documented as commonly-used tactics of warfare in the conflict. Between 2014 and 2017 alone, more than 6,000 children were recruited by armed forces mainly active in Eastern DRC [39]. We hypothesized that higher exposure to war-related trauma would be associated with higher maladjustment, i.e., increased levels of mental health problems and more frequent engagement in criminal behaviour. However, we also hypothesized that these associations would be significantly reduced at higher levels of perceived quality of relationships with caregivers.

## Methods

### Participants

Overall, 315 youth participated in the study. Eighteen participants did not provide information on experiences of war-related events and mental health symptoms, while 29 participants did not have any parents or other caregivers. After excluding these participants, the sample for the present study consisted of 268 youth (164 male, M_age_ = 16.31, SD_age_ = 2.36, range_age_ = 11–25 years). More than half (56.7%, n = 152, 107 male) reported having been a member of an armed group. Sociodemographic characteristics of the study sample are shown in Table 1.

**Table 1:** Descriptive statistics of sociodemographic and other study variables

Age in years, *M* (*SD*, *Min–Max*)	16.31 (2.36, 11–25)
Female gender, *%* (*n*)	38.8 (104)
Years of formal education, *M* (*SD*, *Min–Max*)	6.02 (2.99, 0–13)
Membership in armed group	
Former member, *%* (*n*)	56.7 (152)
Age when joined, *M* (*SD*, *Min–Max*)	12.09 (2.68, 4–18)
Age when left, *M* (*SD*, *Min–Max*)	14.27 (2.40, 7–19)
Number of experienced and witnessed war-related events, *M* (*SD*, *Min–Max*)	13.62 (6.08, 0–22)
Number of perpetrated war-related acts of violence, *M* (*SD*, *Min–Max*)	2.16 (2.97, 0–8)
Posttraumatic stress symptoms, *M* (*SD*, *Min–Max*)	7.56 (5.98, 0–23)
Emotional problems, *M* (*SD*, *Min–Max*)	11.06 (3.85, 1–19)
Behavioural problems, *M* (*SD*, *Min–Max*)	7.41 (3.42, 0–17)
Number of committed criminal acts, *M* (*SD*, *Min–Max*)	1.28 (1.89, 0–9)

### Procedure

The study was part of a larger research project that examined the well-being and social capital of war-affected Congolese youth. Previous studies derived from this project focused on classifying youth based on their trauma exposure and perpetration of violence [13] and on gender differences in youth’s social relationships [59]. In contrast, the present study focus on the moderating role of the perceived quality of relationships with caregivers in the association between youth’s exposure to traumatic experiences and their adjustment.

Participants were recruited with the help of seven local child protection organisations using convenience sampling. After explaining the research purpose and procedures, the administrators of the organisations gave their permission to conduct the study and identified potential participants among the youth engaging with their services, taking into account youth`s capacity to participate and their caregivers’ view. Invited participants received detailed information about study content and procedures and provided informed consent for participation. All the youth who were invited agreed to participate. For underaged participants, additional informed consent was obtained from parents or from the administrators of collaborating organisations if they represented the legal guardians.

Data were collected through individual quantitative structured interviews conducted by three (two female and one male) experienced researchers from Western Europe with a background in social science or clinical psychology and experience in conducting research with war-affected youth including former child soldiers. The interviewers were supported by four local translators (two female and two male) who directly translated the questions from English to Kiswahili, the lingua franca in Eastern DRC, and the participants’ answers from Kiswahili to English. Measures to ensure the quality of the translations included comprehensive theoretical and practical training for the translators prior to the data collection, the availability of written Kiswahili versions of all interview measures translated according to scientific guidelines and the use of standardized instruction and administration procedures in the interviews. Interviews lasted approximately one hour and were conducted in a discrete setting on the premises of the organisations. All the youth except for one completed the interview. Female participants were preferably paired with female interviewers and translators. Given the vulnerability of the study participants and the sensitive nature of the questions, interviewers and translators were trained to provide emotional support to participants who felt distressed during or after the interview and participants could be referred to mental health services if they expressed wish for further support. Participants received a compensation equivalent to 5 USD. The study received ethical approval by the Ethical Committee of Bielefeld University (No. 2018–202). Additional details about the study procedures may be found elsewhere [13, 59].

### Measures

#### Sociodemographic characteristics

Participants answered questions about their gender, age, education, family/household size and their involvement with armed groups (e.g., name of the group).

#### Exposure to war-related trauma and perpetration of war-related violence

A modified version of the Violence, War, and Abduction Exposure Scale (VWAES; [14]) was used to assess lifetime exposure to war-related traumatic events. The VWAES has been used in studies with former child soldiers in the Great Lakes Region of Africa, including the DRC [22]. For this study, the items were slightly adapted to fit the context of the current conflict based on literature research and after consultation with local research staff. The modified version comprised 30 items answered on a binary *yes*(1)/*no*(0) scale. Fourteen items were related to directly experiencing war-related interpersonal violence, e.g., being injured with a weapon, and war-related adversity, e.g., being deprived of food, and eight items referred to witnessing war-related interpersonal violence. These items were summed up to a total score of exposure to war-related trauma (range: 0–22). Eight additional items, which enquired whether participants themselves had perpetrated interpersonal violence against other people in the context of war, were combined into a total score of perpetration of war-related violence (range: 0–8).

#### Perceived quality of relationships with caregivers

An adapted version of the Social Capital Questionnaire for Adolescent Students (SCQ-AS; [43]) was used to assess youth’s perceptions of the quality of their relationships with their parents or caregivers. The measure contained eight statements, such as “I talk to my parents/caregivers when I am having a problem” and “I trust my caregivers/parents”. The wording was adapted depending on who participants identified as their caregivers, e.g., their parents, relatives, or staff at the organisation. Participants indicated the extent to which they agreed with these statements on a 5-point scale ranging from *strongly disagree* (1) to *strongly agree* (5). After recoding responses to one statement (“My parents/caregivers do not understand what I am going through these days”), the responses to all items were combined into a sum score (range: 8 to 40), with higher scores indicating a higher perceived quality of the relationships with parents/caregivers. Internal consistency of this measure was high in the present sample (Cronbach’s alpha = 0.87).

#### Posttraumatic stress symptoms (PTSS)

The International Trauma Questionnaire (ITQ) was used to assess PTSS according to the criteria of the 11th version of the International Classification of Diseases (*ICD-11*). The ITQ has been used previously among war-affected young adults in Northern Uganda [40]. The ITQ includes six items, i.e., two items each for the three PTSD symptom clusters reexperiencing, avoidance and sense of threat, and respondents indicate how much they have been bothered by each symptom in the past month on a 5-point scale from *not at all* (0) to *extremely* (4). Responses were summed up across all items to obtain a total score of symptom severity (range: 0–24). Cronbach`s alpha was 0.81 in the present sample.

#### Emotional and behavioural problems

The self-report version of the Strength and Difficulties Questionnaire (SDQ; [17]) was used to measure youth’s emotional and behavioural problems. The SDQ is a well-established screening tool for mental health problems among children and adolescents and it has been widely used in African contexts [23]. Emotional problems (e.g., “I am often unhappy”, “I am usually on my own”) and behavioural problems (e.g., “I am often accused of lying or cheating”, “I get distracted easily”) are represented by 10 items each. Items are rated on a 3-point scale ranging from not true (0) to certainly true (2). Three items related to behavioural problems and two items related to emotional problems were reverse-coded. Sum scores of emotional problems (range: 0–20) and behavioural problems (range: 0–20) were created by summing up the responses to the respective items. Cronbach’s alpha coefficients in the present sample were 0.65 for the emotional problems subscale and 0.59 for the behavioral problems subscale. This low internal consistency is in line with previous studies in African contexts [23] and may be explained by the heterogeneity of items within the subscales [21].

#### Criminal behaviour

Engagement in criminal acts within the preceding 12 months was assessed using a checklist based on available measures of criminal behaviour [38, 45] and adapted to the study context. The checklist consisted of 10 offending behaviours differing in type (e.g., violent crime, property crime) and severity (e.g., purposely damaging property, killing someone). Only acts that happened outside of the context of armed conflict were considered. Participants indicated whether they had committed a particular act in the past year on a binary *yes*(1)/*no*(0) scale. For the analysis, a sum score (range: 0–10) was created by summing up the responses to all items.

#### Statistical analyses

Analyses were conducted using IBM SPSS Statistics 28. In total, there were 169 missing values (< 0.01%) in the relevant study variables across all participants. For family relationships, missing values were replaced by the mean value of the scale. For all other variables, missing values were considered as indicating an absence of the respective symptom, behaviour or event. In the first step, we calculated descriptive statistics of and bivariate correlations between the study variables. Next, we conducted several moderation analyses using the PROCESS macro to examine the moderating effect of perceived quality of caregiver relationships on the associations between exposure to war-related events and the four outcomes PTSS, emotional problems, behavioural problems and criminal behaviour. In each of the four regression models, we entered exposure to war-related trauma, perceived quality of relationships with caregivers and the interaction of these two variables after mean-centering them as main predictors. A significant interaction was indicative of a moderation effect. We included perpetration of war-related violence for two reasons: first, to examine its independent assciations with the different indicators of youth’s adjustment; second, it served as a marker of youth’s involvement with armed groups since only former members of armed groups reported perpetration and 76% of those reported at least one perpetrated act. In addition, we included age and gender as covariates in each model due to their demonstrated importance for the well-being of war-affected youth (e.g., [4, 48]). Before running the models, we tested the necessary assumptions for linear regression analyses. There were no multivariate outliers, but the boxplot revealed three univariate outliers in the outcome criminal behaviour. As removing these cases did not influence the model results, they were retained in the analyses. Partial regression plots showed that the linearity assumption was met for all four models and Durbin-Watson test statistics indicated the absence of autocorrelation. The variance inflation factor did not exceed 1.82. Hence, we did not need to take multicollinearity into account. However, normality and heteroscedastic assumptions were violated for the models predicting criminal behaviour and PTSS as indicated by normal probability plots and residual plots. Therefore, we used bootstrapped coefficients (5000 samples) and heteroscedasticity-consistent standard errors in these two models. Adjusted R^2^ was used to evaluate the fit of the regression models, indicating the percentage of variance in outcome explained by all predictor variables. For visualising significant interaction effects, we requested values of the outcome variable at average, low (one standard deviation below the mean) and high levels (one standard deviation above the mean) of the independent variable war exposure and the moderator variable perceived quality of relationships with caregivers, respectively. All analyses used two-tailed testing at an α-level of 0.05. However, due to the multiple testing problem, we adjusted the two-tailed α-level for testing the significance of the interaction between exposure to war-related trauma and perceived quality of relationships with caregivers using Bonferroni correction (adjusted *α* = 0.0125).

## Results

**Fig. 1:**
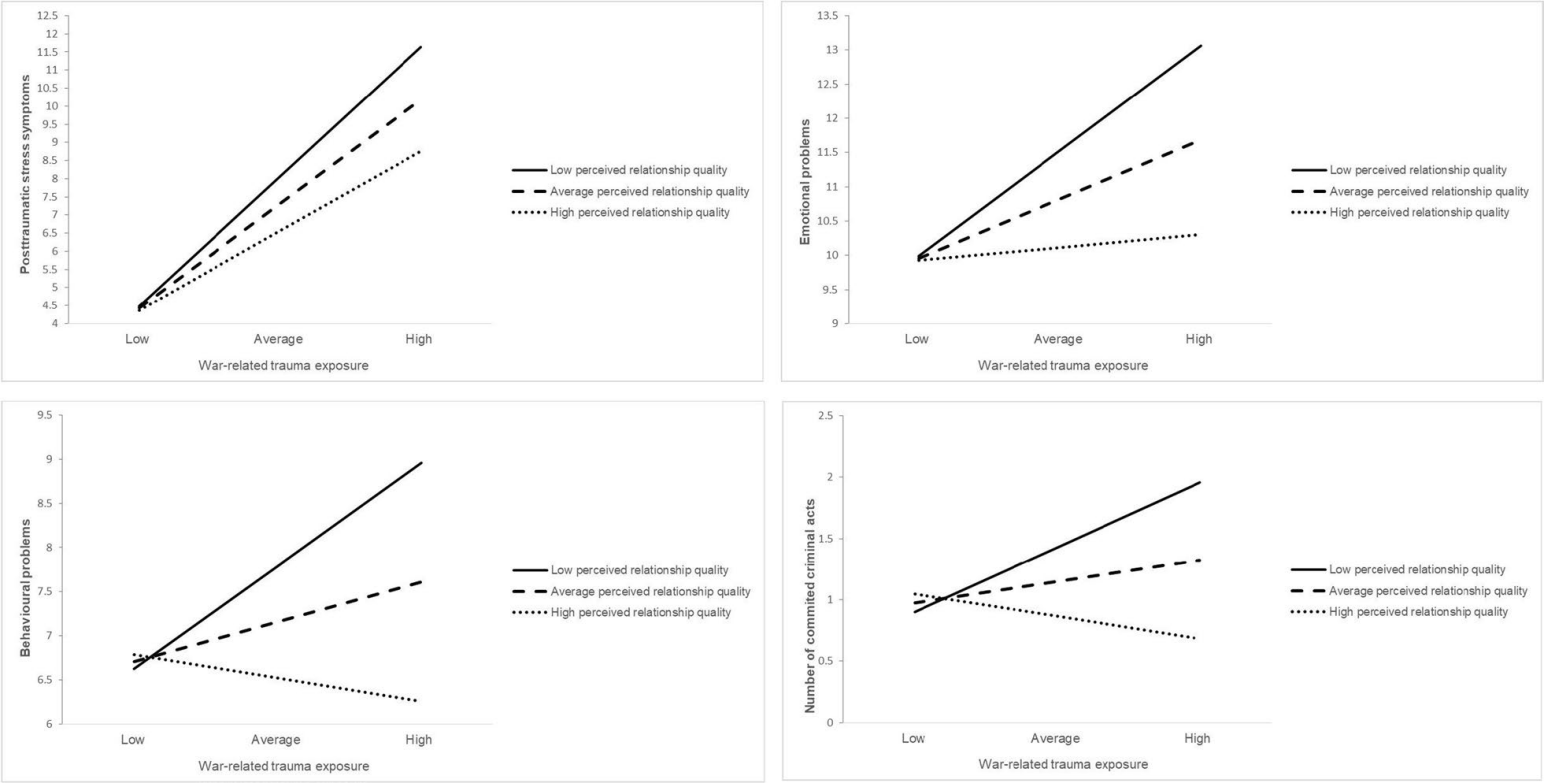
Visualization of moderation effects of perceived quality of relationships with caregivers on the associations between exposure to war-related trauma and posttraumatic stress symptoms (top left), emotional problems (top right), behavioural problems (bottom left) and engagement in criminal behaviour (bottom right)

Descriptive statistics of study variables are shown in Table 1 and bivariate correlations are displayed in Table 2. The results of the four moderation models are presented in Table 3. In the model predicting PTSS, higher exposure to war-related events and more frequent perpetration of war-related violence were significantly and independently related to higher levels of PTSS symptoms. Higher perceived quality of relationships with caregivers was significantly associated with lower levels of PTSS. There was a significant interaction between war exposure and the perceived quality of relationships with caregivers. Although war exposure was significantly positively related to PTSS at all levels of perceived caregiver relationship quality, this association was substantially weaker for higher perceived levels of relationship quality (*b* = 0.36, *t* = 6.18, *p* < 0.001) compared to average (b = 0.48, t = 9.15, *p* < 0.001) and lower levels (*b* = 0.59, *t* = 8.20, *p* < 0.001) of perceived relationship quality (see Fig. 1).

**Table 2:** Bivariate correlations between study variables

	1	2	3	4	5	6	7	8	9
Exposure to war-related traumatic events	–								
Perpetration of war-related violence	0.59***	–							
Perceived quality of relationships with caregivers	− 0.36.***	− 0.35***	–						
PTSS	0.64***	0.54***	− 0.41***	–					
Emotional problems	0.30***	0.23***	− 0.33***	0.36***	–				
Behavioural problems	0.29***	0.36***	− 0.38***	0.39***	0.59***	–			
Criminal behaviour	0.33***	0.50***	− 0.39***	0.37***	0.29***	0.44***	–		
Age	0.27***	0.18**	− 0.05	0.12*	< 0.01	0.03	− 0.03	–	
Gender (1 = male, 0 = female)	0.16*	0.37***	− 0.14*	0.16**	− 0.06	0.17**	0.28***	0.30***	–

Correlations involving gender are Spearman correlations, all other correlations are Pearson correlations

PTSS, posttraumatic stress symptoms

^***^p < 0.001, ^**^p < 0.01, ^*^p < 0.05

**Table 3:** Results of multiple regression analyses

	PTSS^a^				Emotional problems^b^				Behavioural problems^c^				Criminal behaviour^d^			
	B^e^(LL, UL)	SE^f^	t	p	B(LL, UL)	SE	t	p	B^e^(LL, UL)	SE^f^	t	p	B^e^(LL, UL)	SE^f^	t	p
Age	− 0.11(− 0.36, .14)	0.13	−0.86	0.39	− 0.06(− 0.25, 0.14)	0.10	− 0.58	0.56	**− **0.09(**− **0.26, 0.08)	0.08	**− **1.07	0.29	**− .013(− 0.20, − 0.06)**	**0.04**	**− 3.57**	**< 0.001**
Gender	− 0.31(− 1.56, 1.01)	0.66	−0.47	0.64	**-1.18(-2.16, -.21)**	**0.50**	− **2.38**	**0.02**	0.57(-.27, 1.40	0.42	1.34	0.18	**0.50(0.11, 0.91)**	**0.20**	**2.47**	**0.01**
Exposure to war-related trauma	**0.48(0.37, 0.57)**	**0.05**	**9.15**	**< 0.001**	**.14(.05, .23)**	**0.05**	**2.99**	**0.003**	0.07(<-01, .15)	0.09	1.82	0.07	0.03(< **− 0**.01, 0.06)	0.02	1.87	0.06
Perpetration of war-related violence	**0.44(0.16, 0.73)**	**0.14**	**3.06**	**0.003**	0.10(− 0.09, 0.29)	0.10	1.04	0.30	**0.20(0.03, 0.37)**	**0.08**	**2.40**	**0.02**	**0.23(0.11, 0.33)**	**0.05**	**4.18**	**< 0.001**
Perceived quality of relationships with caregivers	− **.009(**− 0**.15,**− 0**.02)**	**0.03**	− **2.78**	**0.006**	− 0**.08(**− **0.14, **− 0**.02)**	**0.03**	− **2.75**	**0.006**	**− 0.07(− 0.12, − 0.02**	**0.03**	**− 2.90**	**0.004**	**− 0.03(− 0.06, − 0.01)**	**0.01**	**− 2.57**	**0.01**
War exposure * Relationship quality	− 0**.01(**− 0**.02, <0.01)**	**0.01**	− **2.84**	**.005**^**g**^	− **0.01(**− **0.02, < **− 0**.01)**	**< .01**	− **2.67**	**0.008**^**g**^	**− 0.01(− 0.02, < − .01)**	**< 0.01**	**− 3.32**	**0.001**^**g**^	**− 0.01(-.01, < − 0.01)**	**< 0.01**	**3.17**	**0.002**^**g**^

Significant parameters are highlighted in bold; PTSS = posttraumatic stress symptoms; ^a^ adj. *R*^2^ = 0.49, *F*(6, 261) = 57.85, *p* < 0.001; ^b^ adj. *R*^2^ = 0.19, *F*(6, 261) = 9.98, *p* < 0.001; ^c^ adj. *R* ^2^ = 0.24, *F*(6, 261) = 13.99, *p* < 0.001; ^d^ adj. *R*^2^ = 0.36, *F*(6, 261) = 13.54, *p* < 0.001; ^e^ coefficients obtained through bootstrapping due to violation of normality assumption; ^f^ heteroscedasticity-consistent standard errors; ^g^ given four tests the Bonferroni-corrected α-level to evaluate significance of the respective coefficients was 0.0125 (0.05/4)

In the model predicting emotional problems, higher exposure to war-related events was significantly associated with higher levels of emotional problems. Higher perceived quality of relationships with caregivers was significantly related to lower levels of emotional problems. A significant interaction indicated moderation of the association between war exposure and emotional problems by the perceived quality of the relationship with caregivers. As visualized in Fig. 1, higher exposure to war-related events was significantly associated with higher levels of emotional problems only at lower (*b* = 0.25, *t* = 3.65, *p* < 0.001) and average levels (*b* = 0.14, *t* = 2.99, *p* < 0.01) of perceived relationship quality, but not at higher levels (*b* = 0.03, *t* = 0.57, *p* = 0.572) of perceived quality of relationships with caregivers.

In the model predicting behavioural problems, more frequent perpetration of war-related violence was significantly related to higher levels of behavioural problems. Higher perceived quality of relationships with caregivers was significantly related to lower levels of behavioural problems. While the association between war exposure and behavioural problems was not significant in the overall model, a significant interaction indicated that the association was moderated by the perceived quality of relationships with caregivers. The association was significant at lower levels (*b* = 0.19, *t* = 3.23, *p* < 0.01) of perceived relationship quality, but not at average (*b* = 0.07, *t* = 1.82, *p* = 0.069) and higher levels (*b* = − 0.04, *t* = − 0.91, *p* = 0.366) of relationship quality (see Fig. 1).

In the model predicting criminal behaviour, more frequent perpetration of war-related violence was significantly associated with more frequent engagement in criminal acts. Higher perceived quality of relationships with caregivers was significantly related to lower levels of criminal behaviour. A significant interaction effect indicated that higher exposure to war-related events was significantly related to more frequent engagement in criminal behaviour only at lower levels (*b* = 0.09, *t* = 3.40, *p* < 0.001) of perceived quality of relationships with caregivers, but not at average (*b* = 0.03, *t* = 1.87, *p* = 0.062) and higher levels (*b* = − 0.03, *t* = − 1.51, *p* = 0.133) of relationship quality (see Fig. 1).

## Discussion

The present study examined whether the perceived quality of relationships with caregivers would mitigate associations between cumulative exposure to war-related trauma and various indicators of adjustment (PTSS, emotional problems, behavioural problems, engagement in criminal behaviour) in war-affected youth in Eastern DRC, more than half of whom were former members of armed groups. As hypothesized, the perceived quality of relationships with caregivers significantly moderated all associations between war-related trauma and youth’s adjustment (mental health problems and criminal behaviour). At high perceived levels of relationship quality, the association between exposure to war-related trauma and PTSS was substantially weaker and the association with emotional problems was no longer significant. In addition, experiencing war-related trauma was significantly related to higher levels of behavioural problems and criminal behaviour only at lower levels of perceived quality of relationships with caregivers, but not at average or high perceived relationship quality.

These findings point to the importance of the family context as a protective social resource for the mental health and functioning of war-affected youth, which is in line with a growing body of research on children’s resilience in the face of armed conflict [6, 48, 55]. This study contributes to the literature by examining the interactive effects of war exposure and perceived quality of relationships with caregivers on a range of mental health outcomes and engagement in criminal behaviour in a sample consisting of youth with and without a history of perpetrating war-related violence. In using this approach, our results extend and corroborate previous findings on the moderating role of perceived quality of relationships with caregivers on the mental health of conflict-affected youth not involved with armed groups (e.g., [32, 51, 53, 56] and on the positive effects of family acceptance on the adjustment of former child soldiers [2, 3, 5, 7]. Given the high psychological burden of our sample and considering the challenges related to detecting significant interaction effects in non-experimental research [61], the findings provide strong evidence for the powerful impact of family relationships on the well-being of youth heavily affected by armed conflict.

Our results suggest that high-quality relationships with caregivers may even offset the ongoing impact of severe war-related trauma on youth’s mental health and functioning. The care, comfort, understanding, and support experienced in close relationships with parents or other adult caregivers appear to be crucial to helping youth process and cope with their traumatic experiences. Having emotionally warm and supportive relationships with caregivers may be particularly important for youth who have been abducted and forced to perpetrate violence to compensate for the deprivation of this critical source of support in a crucial stage of their development [50]. If they feel safe to share their experiences of trauma, both those they witnessed and perpetrated during their time with armed groups, being met with understanding, empathy, and forgiveness by caregivers may be a powerful part of the healing process. Furthermore, caregivers can function as mediators towards the community to actively reduce stigma and support youth’s re-integration, for instance by encouraging community members to take the perspective of affected youth [26].

Notwithstanding its contribution to youth’s resilience, our findings are in line with the notion that the family context can exacerbate the negative impact of war-related trauma on youth’s adjustment [10]. If youth perceived their relationships with their caregivers to be of low quality, higher exposure to war-related trauma was strongly positively related to all four indicators of maladjustment. Low perceived quality of relationships with caregivers indicated the absence of positive interactional qualities such as emotional support and understanding that could mitigate the impact of trauma. However, such relationships may additionally be characterized by hostile relational patterns including abuse and neglect, which have been shown to mediate the impact of war-related trauma on youth`s well-being [10]. Stigma and discrimination due to youth’s (presumed) experiences and actions may transmit from the community to the proximal family level. Family members may also directly blame the youth for things they may have experienced and done. This is supported by significant negative correlations between experiencing and perpetrating war-related violence and the perceived quality of relationships with caregivers in the present study.

The moderating role of perceived quality of relationships with caregivers was especially strong in relation to behavioural problems including hyperactivity and conduct problems as well as to engagement in criminal acts. Since there was no independent effect of cumulative war exposure on these indicators of adjustment, this may be driven more by the negative impact of low-quality relationships with caregivers than the protective effect of relationships of perceived average or high quality. In the absence of protective influences of caregivers on youth’s mental health, trauma-related symptoms may be more likely to be expressed in the form of aggressive and offending behaviours [46]. It is also conceivable that a lack of support and rejection by caregivers may increase youth`s affiliation with deviant peers and subsequent engagement in criminal acts to fulfil their emotional and material needs.

In the overall models, higher levels of experiencing war-related trauma were significantly related to higher levels of PTSS symptoms and emotional problems, while higher levels of perpetrating violence were significantly associated with higher levels of PTSS, behavioural problems and criminal behaviour. This finding mirrors a large body of research documenting a dose–effect of trauma exposure on psychoapthology in war-affected samples of respondents [1, 11, 49]. It is also consistent with previous findings of independent associations between perpetration of war-related violence and PTSS [15] as well as aggression [2, 3, 22]. However, it contrasts with findings of independent effects of perpetration on internalizing symptoms, including depression and anxiety [2, 3, 8, 15], when considering both exposure to and perpetration of war-related violence. This discrepancy may be due to our broader measure of internalizing problems which did not solely focus on clinical symptoms of depression and anxiety. Given the joint consideration of war exposure and perpetration and the inclusion of a range of adjustment outcomes, our findings may indicate that experiencing war-related trauma and perpetration of war-related violence are distinct risk factors for internalizing and externalizing difficulties. Youth who were forced to perpetrate violence as members of armed groups could be especially likely to resort to aggressive and criminal behaviours because these behaviours were adaptive to secure their survival. Being related to both internalizing and externalizing problems, war exposure and perpetration may jointly contribute to PTSS.

This study had a number of limitations. First, the cross-sectional design precludes any directional interpretations of the observed associations. The relations between war exposure (as victim and perpetrator) and indicators of current adjustment may be considered temporally ordered, but the association between relationships with caregivers and adjustment is most likely bidirectional. Longitudinal and experimental designs are needed to examine causal and reciprocal effects. Second, all variables were assessed through youth`s self-reports, which are prone to bias, e.g., related to recall and social desirability. Youth’s perceptions of the quality of their relationships with their caregivers may have been influenced by their current mental health state. Reports by caregivers and observational measures of relationship quality would be helpful in reducing the potential impact of the common-method variance. Third, this study used a convenience sample of youth engaging with local organisations. While this recruitment approach is standard for hard-to-reach and vulnerable populations, it also limits the generalizability of findings and might exclude participants with severe psychological distress. Fourth, we did not take into account youth’s current engagement with mental health and psychosocial support services*.* Fifth, youth without parents or adult caregivers were excluded. However, for such youth, relationships with siblings or friends may replace relationships with caregivers and may have a similarly strong impact on their adjustment. Sixth, the scales representing emotional and behavioural problems exhibited low internal consistency. Although this is in line with previous studies using the SDQ, it may have influenced associations. Seventh, despite their great research experience in the study setting and clinical expertise, we acknowledge that the interviewers from a Western cultural background were likely to be less familiar with specific socio-cultual aspects relevant to this study, e.g., expression of specific symptoms, than local interviewers. However, participants in settings affected by ethnic tensions might also feel more comfortable to share senstive topics with cultural outsiders who may be perceived as more neutral.

The findings have important practical implications because they underscore the importance of family-focused approaches to support the mental health and adjustment of youth affected by armed conflict, in particular those involved with armed groups. As war exposure was significantly positively related to PTSS among youth even at high perceived levels of relationship quality, evidence-based trauma-focused methods to reduce the mental health impact of trauma are indicated. Previous randomized controlled studies have shown positive effects of trauma-focused approaches on the mental health of war-affected youth including former child soldiers, e.g., for trauma-focused cognitive behavioral therapy in the DRC [37] and narrative exposure therapy in Uganda [16].

However, interventions should also actively involve caregivers with the primary aim of strengthening relationships with youth. To achieve this, programs should consider multiple strategies, implemented in a culturally sensitive and appropriate way. Psychoeducation regarding the impact of youth’s traumatic experiences on their mental health and everyday functioning is important to foster understanding and empathy for youth among caregivers. Furthermore, interventions should equip caregivers with strategies to improve family communication and create a supportive atmosphere in which youth feel safe to share their experiences and problems. Caregivers themselves may be suffering from distress and impairment due to their own war-related experiences, which could affect their ability to engage in positive interactions with youth and and adequately respond to their needs. Therefore, it is crucial that family-focused programs also address caregivers` experiences and mental health problems. Joint sessions with caregivers and youth may be particularly useful to practise interactional and coping skills and encourage them to support each other in using these strategies in everyday life. More research is needed on the development, testing and implementation of family systemic intervention approaches in war-torn settings.

## Conclusions

The findings of this study suggest that relationships with caregivers powerfully shape the adjustment of war-affected youth. Close and supportive relationships with caregivers appear to protect youth from the harmful impact of war exposure on their mental health and functioning, whereas relationships characterized by a lack of closeness and emotional warmth seem to exacerbate the effects of war-related traumatic experiences on youth’s well-being. Improving family cohesion and relationships can be an important building block in reintegration and peacebuilding efforts in war-affected regions.

## Data Availability

The data that support the findings of this study are available from the corresponding author upon reasonable request.
